# Carbon- and Binder-Free NiCo_2_O_4_ Nanoneedle Array Electrode for Sodium-Ion Batteries: Electrochemical Performance and Insight into Sodium Storage Reaction

**DOI:** 10.1186/s11671-016-1271-6

**Published:** 2016-02-01

**Authors:** Jong-Won Lee, Hyun-Sup Shin, Chan-Woo Lee, Kyu-Nam Jung

**Affiliations:** New and Renewable Energy Research Division, Korea Institute of Energy Research, 152 Gajeong-ro, Yuseong-gu, Daejeon, 34129 Republic of Korea; Department of Advanced Energy and Technology, Korea University of Science and Technology, 217 Gajeong-ro, Yuseong-gu, Daejeon, 34113 Republic of Korea; Energy Efficiency Research Division, Korea Institute of Energy Research, 152 Gajeong-ro, Yuseong-gu, Daejeon, 34129 Republic of Korea; Department of Chemical and Biomolecular Engineering, Yonsei University, 50 Yonsei-ro, Seodaemun-gu, Seoul, 03722 Republic of Korea

**Keywords:** Sodium-ion battery, Nickel-cobalt oxide, Nanoneedle array, Sodium storage, Conversion reaction

## Abstract

Sodium (Na)-ion batteries (NIBs) have attracted significant interest as an alternative chemistry to lithium (Li)-ion batteries for large-scale stationary energy storage systems. Discovering high-performance anode materials is a great challenge for the commercial success of NIB technology. Transition metal oxides with tailored nanoarchitectures have been considered as promising anodes for NIBs due to their high capacity. Here, we demonstrate the fabrication of a nanostructured oxide-only electrode, i.e., carbon- and binder-free NiCo_2_O_4_ nanoneedle array (NCO-NNA), and its feasibility as an anode for NIBs. Furthermore, we provide an in-depth experimental study of the Na storage reaction (sodiation and desodiation) in NCO-NNA. The NCO-NNA electrode is fabricated on a conducting substrate by a hydrothermal method with subsequent heat treatment. When tested in an electrochemical Na half-cell, the NCO-NNA electrode exhibits excellent Na storage capability: a charge capacity as high as 400 mAh g^−1^ is achieved at a current density of 50 mA g^−1^. It also shows a greatly improved cycle life (~215 mAh g^−1^ after 50 cycles) in comparison to a conventional powder-type electrode (~30 mAh g^−1^). However, the Na storage performance is still inferior to that of Li, which is mainly due to sluggish kinetics of sodiation–desodiation accompanied by severe volume change.

## Background

Recently, sodium (Na)-ion batteries (NIBs) have received considerable attention as a promising alternative to current lithium (Li)-ion batteries (LIBs), mainly due to the abundance of the element Na and its cost-effectiveness [1–4]. In particular, replacing LIBs with NIBs is a potential strategy to fulfill the cost requirements for large-scale stationary energy storage systems. In comparison to Li, however, Na has a larger ionic radius and a higher redox potential, which make electrochemical performance of NIBs inferior to that of LIBs. One of the main challenges for the successful development of NIB technology is thus to find suitable electrode materials that offer excellent Na storage capability [5–9]. Although much research focus has been on designing and synthesizing cathode (positive electrode) materials with high specific capacities, high operating voltages, and long cycle life, little attention has been given to anode (negative electrode) materials for NIBs.

To date, transition metal oxides have been extensively studied as anode materials for use in LIBs due to their high specific capacities delivered via the conversion reaction of oxides with Li [10–22]. The complete reduction of transition metal ions during the lithiation process leads to much higher capacities compared with conventional intercalation materials (e.g., graphite). However, the large volume changes of transition metal oxides during the conversion reaction combined with their low electronic conductivity severely hinder their application in practical LIB systems [5, 10, 14, 21]. A nanostructured electrode designed by tailoring the morphologies and surface structures at a nanoscale has been proposed to alleviate the problems mentioned above by effectively accommodating the strain induced by volume change and providing a short path for charge conduction [12–22]. For instance, several experimental works have demonstrated that Co_3_O_4_ spinels with properly tailored nanostructures deliver high Li storage capacities (ca. 800–1200 mAh g^−1^), and at the same time, they exhibit stable cyclability [12, 13, 16, 20, 21].

A mixed transition metal oxide, NiCo_2_O_4_, is also of significant interest because it exhibits higher electrical conductivity and electrochemical activities toward the conversion reaction with Li in comparison to Co_3_O_4_ [23–27]. As an example, Li et al. reported that the mesoporous NiCo_2_O_4_ anode exhibits a high specific capacity of ~1200 mAh g^−1^ as well as stable cycling performance for ~500 cycles [23]. On the other hand, there are only a few reports on the electrochemical Na storage behavior of NiCo_2_O_4_ for NIBs [28–30]. Alcántara et al. were the first to demonstrate that NiCo_2_O_4_ has the ability to store Na through the conversion reaction similar to that of Li [28]. In their report, however, the NiCo_2_O_4_ powder prepared by precipitation of oxalate precursors delivered only a reversible capacity of 200 mAh g^−1^ in a Na half-cell and displayed a significant capacity decay within 5 cycles. A recent study reported an interesting result showing that NiCo_2_O_4_ nanowires grown on a carbon cloth exhibit enhanced Na storage capability with stable cyclability [29]. This indicates that, as shown previously in the studies on LIBs, the controlled nanostructural engineering of NiCo_2_O_4_ could be an effective approach to improving Na storage performance.

Here, we report a nanostructured NiCo_2_O_4_ anode for NIBs, i.e., a carbon-, binder-free (oxide-only) NiCo_2_O_4_ nanoneedle array (NCO-NNA) deposited on a conducting substrate. In addition to the feasibility study of NCO-NNA as an anode for NIBs, we provide an in-depth structural and electrochemical analysis on the Na storage reaction (sodiation and desodiation) in the nanostructured oxide-only electrode. The spinel-type NCO-NNA electrode was directly grown on a conducting Ni substrate by a hydrothermal method without using any conducting carbon or binders. The electrochemical Na storage behavior of NCO-NNA was examined and compared with that of Li. Particularly, the conversion reaction of NCO-NNA with Na was investigated using ex situ structural and chemical analyses during the sodiation–desodiation processes, and then, the performance difference of NCO-NNA in Na and Li half-cells was discussed based on the reaction pathways involved in Na and Li storage.

## Methods

### Material Preparation

A spinel-type NCO-NNA was directly deposited on a conducting substrate using a hydrothermal method combined with post-heat treatment. The requisite metal precursors (Co(NO_3_)_2_ · 6H_2_O and Ni(NO_3_)_2_ · 6H_2_O) and urea (CO(NH_2_)_2_) were dissolved in deionized water, and then, the resulting solution was transferred to a Teflon-lined stainless steel autoclave. A nickel foam substrate was placed in the solution, and the autoclave was kept at 120 °C for 9 h. During the hydrothermal process, a mixed metal hydroxide was formed on the Ni substrate. The hydroxide-deposited substrate was thoroughly washed with ethanol and water, then dried under vacuum at 80 °C, and finally heat-treated in air at 350 °C for 3 h to convert the metal hydroxide to NiCo_2_O_4_. The weight of NiCo_2_O_4_ on the Ni substrate was 2.8 mg cm^−2^. For comparison, NiCo_2_O_4_ powder (NCO-P) was also obtained under the same hydrothermal and heat treatment conditions in the absence of the Ni foam substrate.

### Material Characterization

Phase and crystal structure analysis was conducted with an automated HPC-2500 X-ray diffractometer (Gogaku) using Cu *K*_α_ radiation (*λ* = 1.5405 Å). The morphology, microstructure, and composition of the synthesized samples were examined by scanning electron microscopy (SEM, Hitachi S4700) and transmission electron microscopy (TEM, TECNAI G2 F30S-Twin) in conjunction with energy-dispersive X-ray spectroscopy (EDS). X-ray photoelectron spectroscopy (XPS) was performed using a Thermo MultiLab 2000 system with a monochromatic Al *K*_α_ X-ray source. Brunauer–Emmett–Teller (BET) surface area was determined from N_2_ sorption isotherms by using a BEL-SORP mini system.

### Electrochemical Experiments

Electrochemical experiments were conducted using a coin-type cell (CR2032). The NCO-NNA on the Ni substrate was directly used as the working electrode for both Na and Li half-cells. For a Na half-cell, a Na metal (Aldrich) and 1 M NaClO_4_ in propylene carbonate (PC) with 5 wt.% fluoroethylene carbonate (FEC) were employed as the counter electrode and electrolyte, respectively. On the other hand, the Li half-cell was made of a Li metal and 1 M LiPF_6_ in ethylene carbonate (EC)/diethyl carbonate (DEC) (1:1 in volume). The separator was a glass fiber sheet. All of the cells were assembled in a glove box filled with purified Ar gas. The galvanostatic charge–discharge experiments were performed with a Maccor Series 4000 at various current densities in a voltage range of 0.01–3.0 V vs. Na/Na^+^ or Li/Li^+^. Electrochemical impedance spectra were obtained by using a Zahner IM6 with an *ac* amplitude of 5 mV_rms_ on an open-circuit voltage during a frequency sweep from 10^5^ Hz down to 10^−2^ Hz.

## Results and Discussion

### Morphological and Physicochemical Characteristics of NiCo_2_O_4_ Nanoneedle Array (NCO-NNA)

In this work, a spinel-type NCO-NNA was directly grown on a Ni substrate without using any conducting carbon or binders. When used as an electrode for NIBs, this oxide-only nanostructured design would offer the following advantages over conventional composite electrodes made of large particle agglomerates: (i) the nanoneedle architectures synthesized here provide an increased number of active sites for the electrochemical reaction, resulting in improved Na storage capability; and (ii) one can avoid any complications arising from inactive materials (conducting carbon and binders) and thus probe exclusively the physicochemical changes of the electrode induced by electrochemical sodiation and desodiation.

**Fig. 1:**
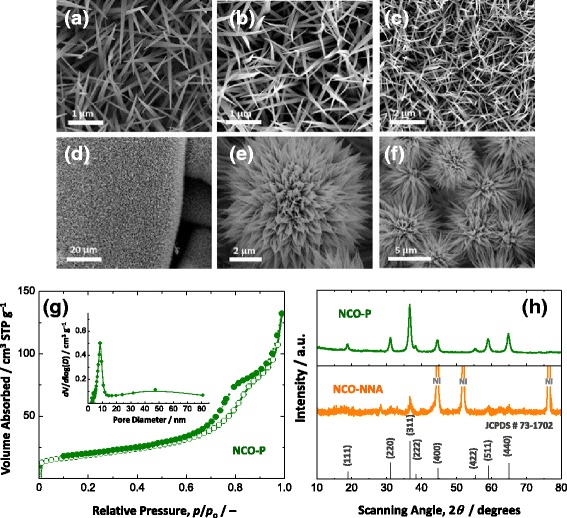
SEM micrographs of **a** the Ni-Co hydroxide nanoneedle array produced from hydrothermal synthesis and **b**–**d** the Ni-Co oxide nanoneedle array (NCO-NNA) and **e**, **f** Ni-Co oxide powder (NCO-P) obtained upon post-heat treatment. **g** N_2_ adsorption-desorption isotherms and corresponding pore size distribution curve (*inset*). **h** XRD patterns for NCO-NNA and NCO-P

The NCO-NNA electrode was fabricated by a two-step synthesis process: first, the mixed Ni-Co hydroxide was formed via the hydrothermal reaction of metal ions with ammonium and hydroxyl ions released by the hydrolysis of urea [26], and then, it was thermally transformed to the spinel-type metal oxide (NiCo_2_O_4_) at 350 °C. Figure 1 shows SEM images of the metal hydroxide (Fig. 1a) produced from the hydrothermal synthesis and the corresponding metal oxide (Fig. 1b–d) obtained upon post-heat treatment in air. The SEM analysis indicates that the unique morphological characteristics of NCO-NNA originated from the one-dimensional (1D) growth of the metal hydroxide during hydrothermal synthesis, and the post-heat treatment in air caused no significant morphological changes. As shown in Fig. 1b–d, the oxide structure exhibits a nanoneedle-like morphology (<100 nm in diameter), and numerous nanoneedles uniformly cover the entire surface of the Ni substrate, producing an NCO-NNA electrode with highly porous architectures. The NiCo_2_O_4_ powders (NCO-P) (Fig. 1e, f) synthesized under the same hydrothermal and heat treatment conditions in the absence of the Ni foam substrate have an urchin-like morphology with nanoneedles vertically grown on a spherical core. The analyses of the nitrogen adsorption-desorption isotherms and corresponding pore size distribution curve (Fig. 1g) indicate that NCO-P has a mesoporous structure and its BET surface area was 66 m^2^ g^−1^. The structural information of NCO-NNA was obtained by XRD analysis as shown in Fig. 1h. All of the diffraction peaks can be successfully indexed to the cubic spinel NiCo_2_O_4_ phase (JCPDS No. 73–1702) without noticeable impurity phases. The XRD pattern of NCO-NNA is in good agreement with that of NCO-P. It seems that the relatively broader diffraction peaks of NCO-NNA in comparison to those of NCO-P are largely due to the presence of smaller nanocrystals in NCO-NNA as well as a relatively smaller weight of the oxide deposited on the Ni substrate.

**Fig. 2:**
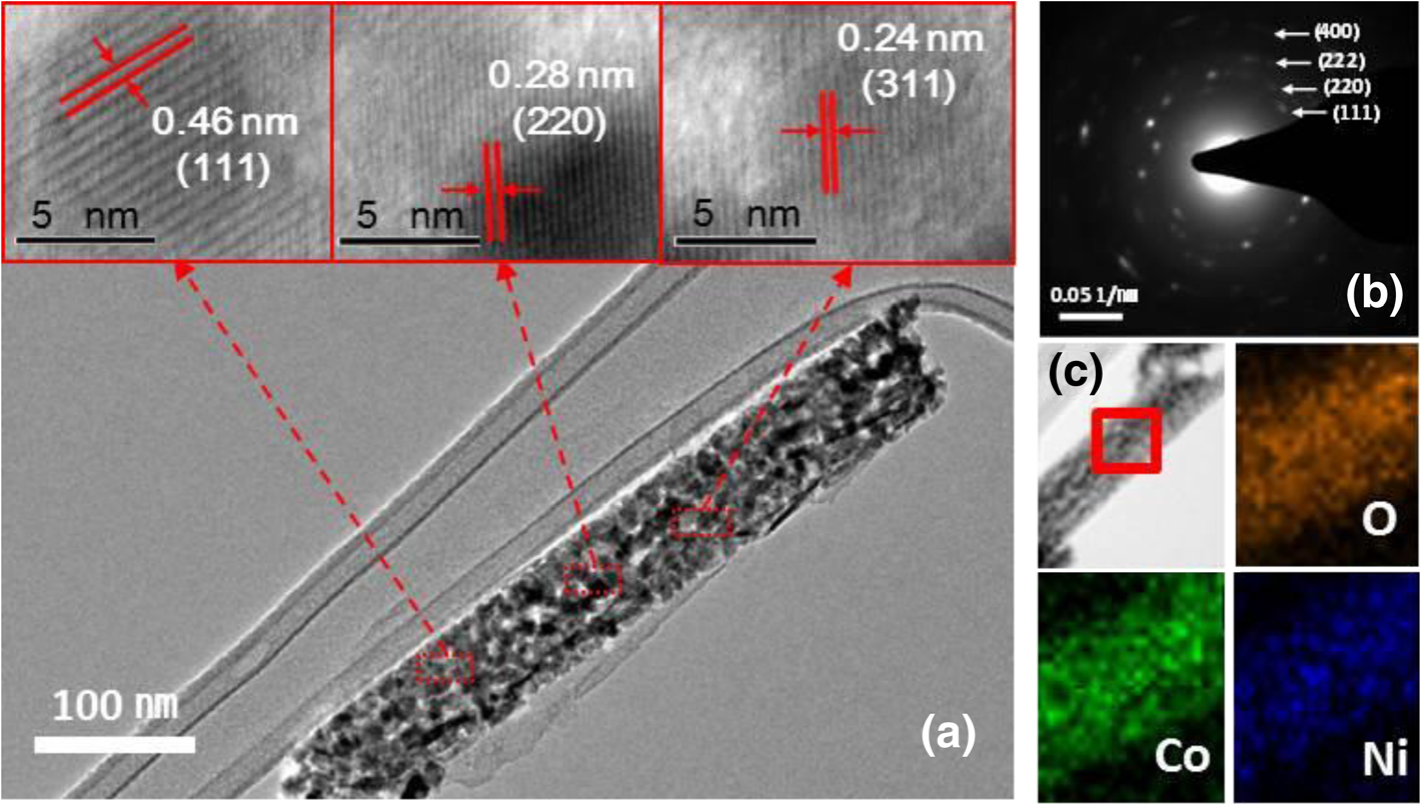
**a** TEM micrographs and **b** SAED pattern for NCO-NNA. **c** EDS mapping of O, Co, and Ni in NCO-NNA

**Fig. 3:**
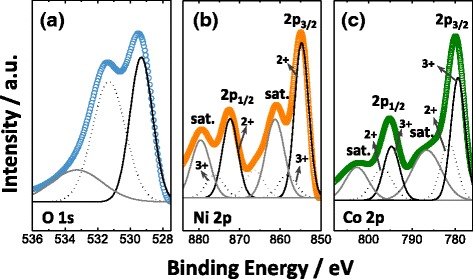
XPS spectra of **a** O 1s, **b** Ni 2p, and **c** Co 2p for NCO-NNA

The TEM micrographs in Fig. 2a confirm that NCO-NNA consists of polycrystalline tiny grains with sizes of ca. 10–30 nm and mesoscale pores among nanocrystals. The lattice fringes with *d*-spacing values of 0.24, 0.28, and 0.46 nm are assigned to the (311), (220), and (111) planes of cubic spinel NiCo_2_O_4_, respectively. Furthermore, the selected area electron diffraction (SAED) pattern of Fig. 2b displays the characteristic diffraction rings corresponding to the (111), (220), and (222) planes of NiCo_2_O_4_, which is well consistent with the XRD result in Fig. 1g. The EDS analysis (Fig. 2c) confirmed the uniform distribution of Ni, Co, and O elements throughout the nanoneedle. The surface composition and oxidation states of NCO-NNA were further investigated by XPS, and the results are presented in Fig. 3a–c. The XPS spectrum for the O 1s region (Fig. 3a) consists of three component curves: the low binding energy peak at ~529.3 eV is ascribed to the metal-oxygen bond; the binding energy peak at ~531.3 eV is associated with defects, contaminants, and surface species (e.g., hydroxyls and chemisorbed oxygen); and the high binding energy peak at ~533.2 eV originates from the adsorbed water species [23, 27]. The Ni 2p spectrum (Fig. 3b) could be well fitted by considering the spin-orbit doublet characteristics of Ni^2+^ and Ni^3+^ with satellite peaks. Similarly, the Co 2p spectrum (Fig. 3c) was deconvoluted into the characteristic curves of Co^2+^ and Co^3+^. According to the XPS results, the electron couples of Ni^3+^/Ni^2+^ and Co^3+^/Co^2+^ coexisted in NCO-NNA and the average oxidation states of Ni and Co were ~2.25 and ~2.64, respectively, which agree well with the earlier report [23].

### Comparative Electrochemical Study on Li and Na Storage Performance of NCO-NNA

**Fig. 4:**
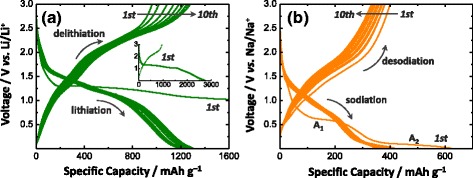
Discharge–charge profiles of NCO-NNA electrodes in **a** Li and **b** Na half-cells measured at a current density of 50 mA g^−1^ during the initial 10 cycles

The mechanism of Li storage in NiCo_2_O_4_ involving a conversion reaction has been well established by previous experimental and theoretical studies [23–27, 29]. It would be thus useful to examine Li storage behaviors of NCO-NNA that can provide benchmark data for a Na storage study. Figure 4a shows the discharge (lithiation) and charge (delithiation) profiles of the NCO-NNA electrode measured for the first 10 cycles at a constant current density of 50 mA g^−1^. The initial discharge curve exhibited a distinct voltage plateau at ~1.25 V vs. Li/Li^+^, followed by a monotonous voltage decrease to 0.01 V vs. Li/Li^+^. The subsequent charge curve displayed a continuous voltage increase with progressing delithiation, resulting in large voltage gaps during the discharge–charge cycle. Such a large voltage hysteresis is typical of the oxide-based electrodes that undergo the conversion reaction with Li [5, 14, 21]. The initial discharge capacity of NCO-NNA in the Li half-cell was estimated to be 2744 mAh g^−1^ (24.6 mol Li in NiCo_2_O_4_). The higher capacity compared to the theoretical value (891 mAh g^−1^ and 8 mol Li) is likely to be due to the interfacial Li storage at the highly mesoporous nanoneedles and the solid–electrolyte interphase (SEI) formation [14, 23, 25, 29]. Furthermore, a low Coulombic efficiency of ~35 % during the first cycle may have resulted from the irreversible formation of SEI and the incomplete decomposition of Li_2_O species during the initial discharge and charge processes, respectively. Upon subsequent cycling, the NCO-NNA electrode exhibited a more reversible lithiation–delithiation behavior, delivering discharge capacities of 1180–1300 mAh g^−1^ (10.6–11.6 mol Li) and charge capacities of 1020–1280 mAh g^−1^ (9.2–11.5 mol Li).

As a next step, a Na half-cell was constructed using NaClO_4_ in PC with FEC as the electrolyte and tested with the NCO-NNA working electrode, and the discharge (sodiation) and charge (desodiation) curves obtained for the first 10 cycles are presented in Fig. 4b. According to the previous studies [31, 32], FEC plays a beneficial role in improving the structural integrity of anodes in Na half-cells by inducing the formation of stable SEI layers in carbonate-based electrolytes. The electrode was discharged to a cutoff voltage of 0.01 V vs. Na/Na^+^ and then recharged to 3.0 V vs. Na/Na^+^. The NCO-NNA electrode delivered 621 and 400 mAh g^−1^ (Coulombic efficiency ~64 %) during the first discharge and charge processes, respectively. It is seen that, while a single plateau appeared for lithiation, two distinct plateau regions were observed for the sodiation reaction of NCO-NNA: (i) a potential plateau (denoted by *A*_1_) at ~0.6 V vs. Na/Na^+^; and (ii) a potential plateau (denoted by *A*_2_) at ~0.1 V vs. Na/Na^+^ (~310 mAh g^−1^). During subsequent discharge–charge cycles, the NCO-NNA electrode showed improved reversibility with discharge–charge capacities of ~400 mAh g^−1^ and Coulombic efficiency of ~91 %.

**Fig. 5:**
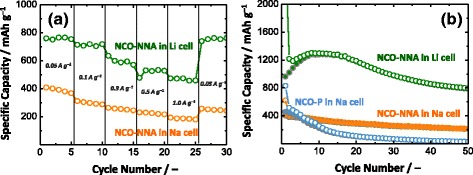
Comparison of **a** rate capability and **b** cycle performance (at 50 mA g^−1^) of NCO-NNA in Li and Na half-cells. The result for NCO-P was also presented in **b**

The rate capability and cycling performance of NCO-NNA were examined in both Li and Na half-cells. The NCO-NNA electrode exhibited excellent rate capability in the Li half-cell; in particular, it retained a high charge capacity of 475 mAh g^−1^ at 1.0 A g^−1^, which was much higher than that of the Na half-cell (187 mAh g^−1^) as shown in Fig. 5a. The cycling performance of NCO-NNA in Li and Na half-cells are illustrated in Fig. 5b. The NCO-NNA electrode in the Li half-cell showed a charge capacity retention of ~82 % during 50 cycles. As has been suggested in previous studies [23, 25], the capacity increase observed during the initial 10 cycles was mainly ascribed to the activation processes, such as the growth of polymer/gel-like layers and/or the restoration of electrochemically less active Li_2_O species. In the Na half-cell, on the other hand, the charge capacity was found to monotonously decrease with cycling, delivering only 215 mAh g^−1^ at the 50th cycle (~56 % capacity retention). Interestingly, we noted that, when tested in the Na half-cell, the NCO-NNA electrode exhibits a considerably improved cyclability in comparison to NCO-P electrode (mixed with 10 wt.% conducting carbon and 10 wt.% PVdF binder). The unique nanoarchitecture of NCO-NNA seems to play a beneficial role in improving the cycle life: (i) the mesoporous nanoneedle structure provides a large number of the active reaction sites while facilitating mass transport through the porous 1D structure; and (ii) NiCo_2_O_4_ is in direct contact with a current collector, thereby reducing the contact resistance between them. This report provides a promising strategy to designing oxide-based anodes with controlled nanoarchitectures for high-performance NIBs. Comprehensive studies should be conducted to find optimum combinations of electrolyte salts and solvents that can work with the NCO-NNA anode and thus to further improve the cycle stability of NIBs.

### Remarks on the Lithiation and Sodiation Reactions of NCO-NNA

It is generally agreed that NiCo_2_O_4_ converts to metallic (Ni and Co) nanoparticles and Li_2_O (Eq. (1)) during the first discharge (lithiation) process, and then, the charge–discharge cycles proceed via the conversion reactions between Ni and NiO (Eq. (2)) and between Co and Co_3_O_4_ (Eqs. (3) and (4)) involving the decomposition and formation of Li_2_O [23–27, 29]:

(i) first discharge (lithiation)

1 $$ {\mathrm{NiCo}}_2{\mathrm{O}}_4 + 8\left({\mathrm{Li}}^{+}+{\mathrm{e}}^{-}\right)\ \to\ \mathrm{N}\mathrm{i} + 2\mathrm{C}\mathrm{o} + 4{\mathrm{Li}}_2\mathrm{O} $$

(ii) first charge (delithiation) and subsequent cycles

2 $$ \mathrm{N}\mathrm{i} + {\mathrm{Li}}_2\mathrm{O}\ \leftrightarrow\ \mathrm{N}\mathrm{i}\mathrm{O} + 2\left({\mathrm{Li}}^{+} + {\mathrm{e}}^{-}\right) $$

3 $$ 2\mathrm{C}\mathrm{o} + 2{\mathrm{Li}}_2\mathrm{O}\ \leftrightarrow\ 2\mathrm{C}\mathrm{o}\mathrm{O} + 4\left({\mathrm{Li}}^{+} + {\mathrm{e}}^{-}\right) $$

4 $$ 2\mathrm{C}\mathrm{o}\mathrm{O} + 2/3{\mathrm{Li}}_2\mathrm{O}\ \leftrightarrow\ 2/3{\mathrm{Co}}_3{\mathrm{O}}_4 + 4/3\left({\mathrm{Li}}^{+} + {\mathrm{e}}^{-}\right) $$

**Fig. 6:**
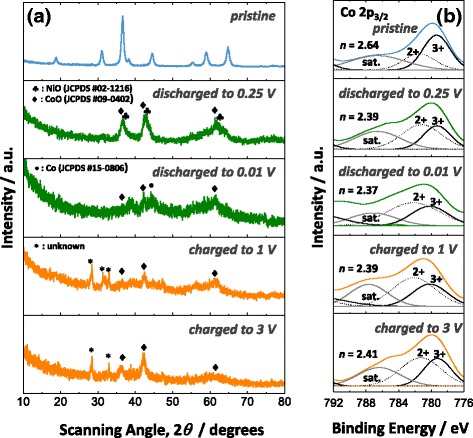
**a** XRD patterns for NCO-P and **b** Co 2p_3/2_ XPS spectra for NCO-NNA obtained at different states of discharge and charge during the first cycle

Having noticed the considerable difference in electrochemical performance of NCO-NNA in Li and Na half-cells, we investigated the sodiation and desodiation behaviors by using ex situ XRD and XPS analyses. Figure 6a shows the ex situ XRD results obtained at different states of discharge and charge during the first cycle. It should be noted that the NCO-P electrode was used for ex situ XRD analysis to detect enhanced diffraction peaks and hence to acquire clear structural information on the discharged/charged products. When the NCO-P electrode was discharged, crystalline peaks corresponding to the NiO and CoO phases were observed at 0.25 V vs. Na/Na^+^ (discharged beyond plateau *A*_1_). This means that plateau *A*_1_ at ~0.6 V vs. Na/Na^+^ is associated with the dissociation reaction of NiCo_2_O_4_ into NiO and CoO intermediates. When further discharged to 0.01 V vs. Na/Na^+^ (discharged beyond plateau *A*_2_), the crystalline metal oxide phases were almost destroyed, while the weak and broad peaks for the CoO and metallic cobalt were vaguely observed, indicating the partial reduction reaction of CoO to Co at plateau *A*_2_ at ~0.1 V vs. Na/Na^+^. The incomplete reduction of CoO to Co might be responsible for the lower initial discharge capacity (622 mAh g^−1^) of NCO-NNA compared to the theoretical value (891 mAh g^−1^). During the initial charge process, the XRD patterns only displayed the characteristic peaks for the CoO phase at 1.0 V vs. Na/Na^+^ and 3.0 V vs. Na/Na^+^ without any signs of the Co_3_O_4_ formation, indicating that the major product formed upon charging is CoO.

The number of the average oxidation state (*n*) of Co was estimated by the ex situ XPS analysis of NCO-NNA at various states of discharge and charge for a further analysis of the structural information on the cobalt oxide species identified by the ex situ XRD analysis. As shown in Fig. 6b, the Co 2p_3/2_ spectra were deconvoluted to determine the *n* value by considering the spin-orbit doublet characteristics of Co^2+^ and Co^3+^ with satellite peaks. During the first discharge process, the *n* value decreased from 2.64 (pristine NCO-NNA) to 2.39 at 0.25 V vs. Na/Na^+^ (discharged beyond plateau *A*_1_), and further diminished to 2.37 at 0.01 V vs. Na/Na^+^ (discharged beyond plateau *A*_2_), indicating an increased amount of Co^2+^ species upon sodiation. After charging to 3.0 V vs. Na/Na^+^, the value of *n* slightly increased to 2.41, which was still similar to that for NCO-NNA discharged to 0.25 V vs. Na/Na^+^. In addition to the XRD results (Fig. 6a), these XPS data provide a clear evidence supporting that, unlike the case of lithiation, the reduced Co species would not convert back to Co_3_O_4_, but rather it is oxidized to CoO during the desodiation process. Based on the XRD and XPS analyses combined with galvanostatic discharge–charge measurements, the conversion reaction of the NCO-NNA electrode in the Na half-cell may be described as follows:

(i) first discharge (sodiation)

5 $$ {\mathrm{NiCo}}_2{\mathrm{O}}_4 + 2\left({\mathrm{Na}}^{+} + {\mathrm{e}}^{-}\right)\to \mathrm{N}\mathrm{i}\mathrm{O} + 2\mathrm{C}\mathrm{o}\mathrm{O} + {\mathrm{Na}}_2\mathrm{O} $$

6 $$ \mathrm{N}\mathrm{i}\mathrm{O} + 2x\left({\mathrm{Na}}^{+} + {\mathrm{e}}^{-}\right)\to \left(1\hbox{--} x\right)\mathrm{N}\mathrm{i}\mathrm{O} + x\mathrm{N}\mathrm{i} + x{\mathrm{Na}}_2\mathrm{O}\kern0.5em \left(0 < x<1\right) $$

7 $$ 2\mathrm{C}\mathrm{o}\mathrm{O} + 4y\left({\mathrm{Na}}^{+} + {\mathrm{e}}^{-}\right)\to \left(2\hbox{--} y\right)\mathrm{C}\mathrm{o}\mathrm{O} + y\mathrm{C}\mathrm{o} + 2y{\mathrm{Na}}_2\mathrm{O}\kern0.5em \left(0 < y < 2\right) $$

(ii) first charge (desodiation) and subsequent cycles

8 $$ \left(1\hbox{--} x\right)\mathrm{N}\mathrm{i}\mathrm{O} + x\mathrm{N}\mathrm{i} + x{\mathrm{Na}}_2\mathrm{O}\leftrightarrow \mathrm{N}\mathrm{i}\mathrm{O} + 2x\left({\mathrm{Na}}^{+} + {\mathrm{e}}^{-}\right)\kern0.75em \left(0 < x<1\right) $$

9 $$ \left(2\hbox{--} y\right)\mathrm{C}\mathrm{o}\mathrm{O} + y\mathrm{C}\mathrm{o} + 2y{\mathrm{Na}}_2\mathrm{O}\leftrightarrow 2\mathrm{C}\mathrm{o}\mathrm{O} + 4y\left({\mathrm{Na}}^{+} + {\mathrm{e}}^{-}\right)\kern0.75em \left(0 < y < 2\right) $$

**Fig. 7:**
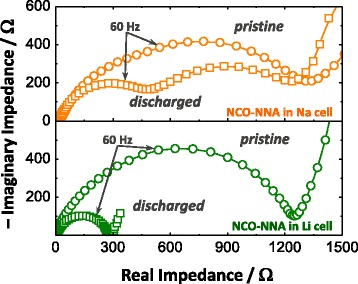
*Ac* impedance spectra of the NCO-NNA electrodes in Li and Na half-cells measured before and after the first discharging step

As described in Eqs. (1)–(9), the conversion reaction of NCO-NNA with Li or Na involves the formation and decomposition of Li_2_O or Na_2_O, respectively. That is, Li_2_O or Na_2_O is formed at the expense of the oxide, and its decomposition is accompanied by the formation of the prior oxide. According to the experimental results presented here, the sodiation–desodiation reactions of NCO-NNA proceed via the conversion mechanism, analogous to those of lithiation–delithiation, but with a lower degree of conversion. This may account for the inferior electrochemical performance of NCO-NNA in the Na half-cell, as shown in Figs. 4 and 5. It is likely that the less negative Gibbs free energy of Na_2_O formation (−375.8 kJ mol^−1^) in comparison to that of Li_2_O (−561.2 kJ mol^−1^) leads to the incomplete reduction of the oxide into the metals (Ni and Co) upon sodiation. Moreover, the larger molar volume of Na_2_O (27.3 cm^3^ mol^−1^) than that of Li_2_O (14.8 cm^3^ mol^−1^) results in more severe volume changes during the sodiation–desodiation cycle, which destroy the integrity of NCO-NNA microstructures and thus reduce the kinetics and reversibility of the conversion reaction with Na [5, 28]. The latter can be further supported by the *ac* impedance results. The *ac* impedance analysis (Fig. 7) revealed that the pristine NCO-NNA electrodes had the similar values (ca. 1280–1380 Ω) of the interfacial polarization resistances in Li and Na half-cells. While the value of the interfacial polarization resistance drastically decreased from ca. 1280 Ω to ca. 280 Ω upon lithiation, the total value of the interfacial polarization resistances of the sodiated electrode (due to the contributions of the SEI layer and charge-transfer reaction) remained comparable to that of the pristine one. The high film resistance may be ascribed to the formation of stable yet resistive SEI layers containing FEC-derived NaF in the Na half-cell [31, 32]. More importantly, the higher charge-transfer resistance in the Na half-cell than in the Li half-cell indicates more sluggish kinetics of the conversion reaction with Na.

## Conclusions

In summary, we developed a carbon- and binder-free NiCo_2_O_4_ nanoneedle array for use as an NIB anode, which was fabricated on a conducting substrate by the hydrothermal method with subsequent heat treatment. When tested in the Na half-cell, the NCO-NNA electrode exhibits a considerably improved cycle performance over the conventional composite electrode. The enhanced performance of NCO-NNA is mainly due to the unique electrode nanoarchitecture, which provides an increased number of active sites for the Na storage while facilitating mass transport through the porous 1D structure and reducing the contact resistance with current collector. However, the comparative electrochemical study on Li and Na storage revealed that the Na storage performance of NCO-NNA is inferior to that of Li in terms of capacity, cycling stability, and rate capability, which could be explained by the reduced kinetics and reversibility of the conversion reaction with Na involving Na_2_O formation and decomposition.
